# Supplementary material for: Radical-Generating Activity, Phagocytosis, and Mechanical Properties of Four Phenotypes of Human Macrophages
Source: Int J Mol Sci. 2024 Feb 3;25(3):1860. doi: 10.3390/ijms25031860 (PMC10855323; doi:10.3390/ijms25031860)
Supplement: Supplementary file 1 [file ijms-25-01860-s001.zip › ijms-2784051-supplementary.pdf]

*Supplementary Material for*

# *Radical-generating activity, phagocytosis, and mechanical properties of four phenotypes of human macrophages*

***[1,2]Suleimanov Sh.K., [3]Efremov Yu.M., [1,2]Klyucherev T.O., [3]Salimov E.L., [3]Ragimov A.A., [1,4]Timashev P.S., [1]Vlasova I.I.****

***[1]Institute for Regenerative Medicine,***
***[2]Laboratory of Clinical Smart Nanotechnologies,***
***[3]Laboratory blood transfusion complex,***
***[4]World-Class Research Center "Digital biodesign and personalized healthcare“,***
***I. M. Sechenov First Moscow State Medical University (Sechenov University), Moscow, 119991 Russia***

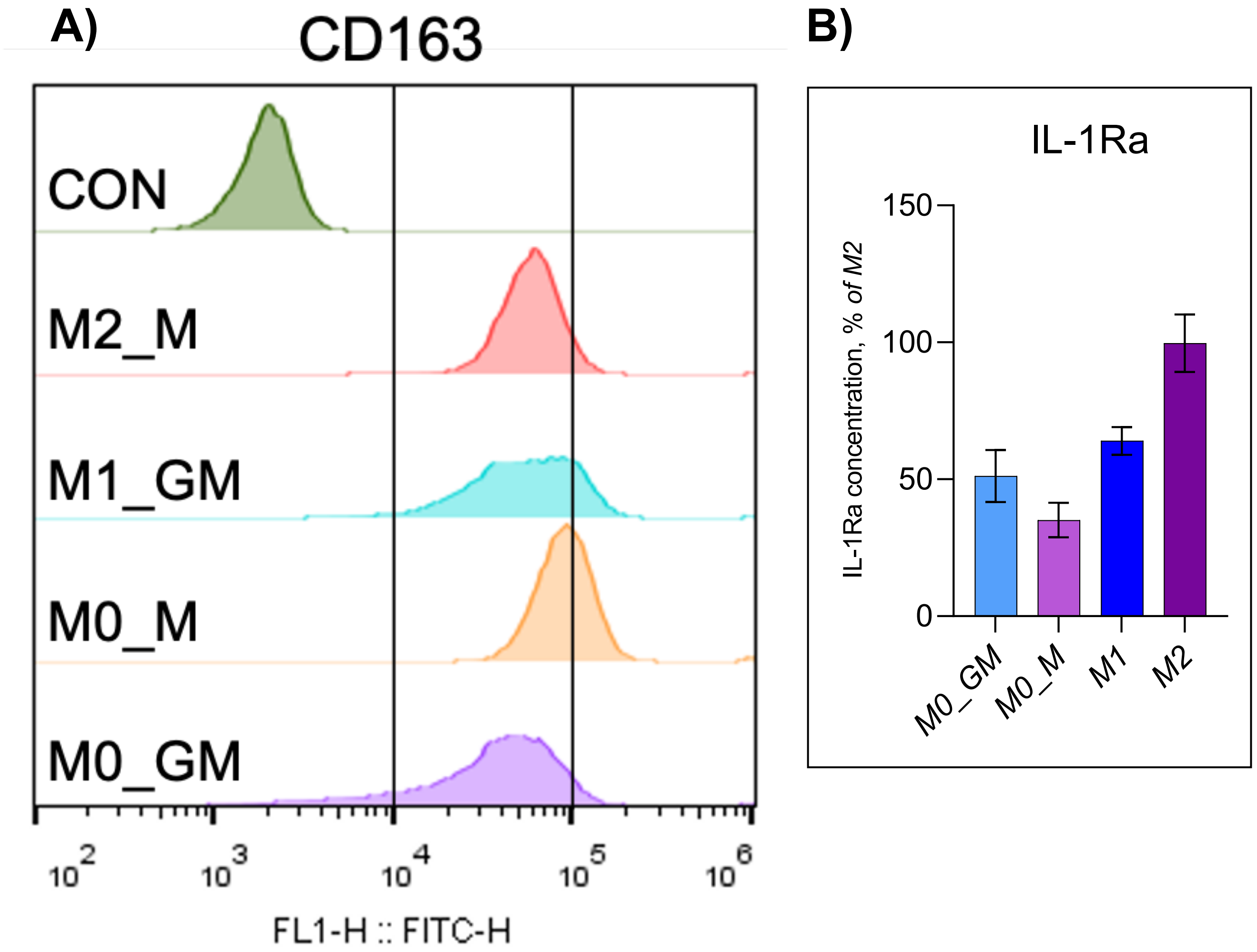


**Figure S1.Typical markers of M2 macrophages.**
**A)** Expression of CD163 on the membrane of different phenotypes of MDM.
Flow cytometry histograms presenting fluorescence intensity of M0, M1, and M2 macrophages incubated with primary anti-CD163 and secondary anti-Rb-Alexa Fluor 488 antibodies. Half of the cells cultured with GM-SCF (50 ng/ml) (M0_GM) was treated by LPS (10 ng/ml) and IFN-γ (50 ng/ml) (M1_GM), cells cultured with M-CSF (50 ng/ml) (M0_M) were treated by IL-4 (20 ng/ml) (M2_M). CON stands for unstained cells. In two days of incubation, macrophages were detached by accutase and stained with primary anti-CD163 rabbit monoclonal antibodies for 30 min in dark at 4°C. After incubation, cells were washed twice and stained with secondary anti-rabbit Alexa Fluor 488 antibodies for 30 min in dark at 4°C. Cells were washed with flow buffer prior to measurements.
**B)** Concentration of IL-1Ra in MDM cultivation media (% of M2) was measured with ELISA.
The results are typical of two independent experiments.

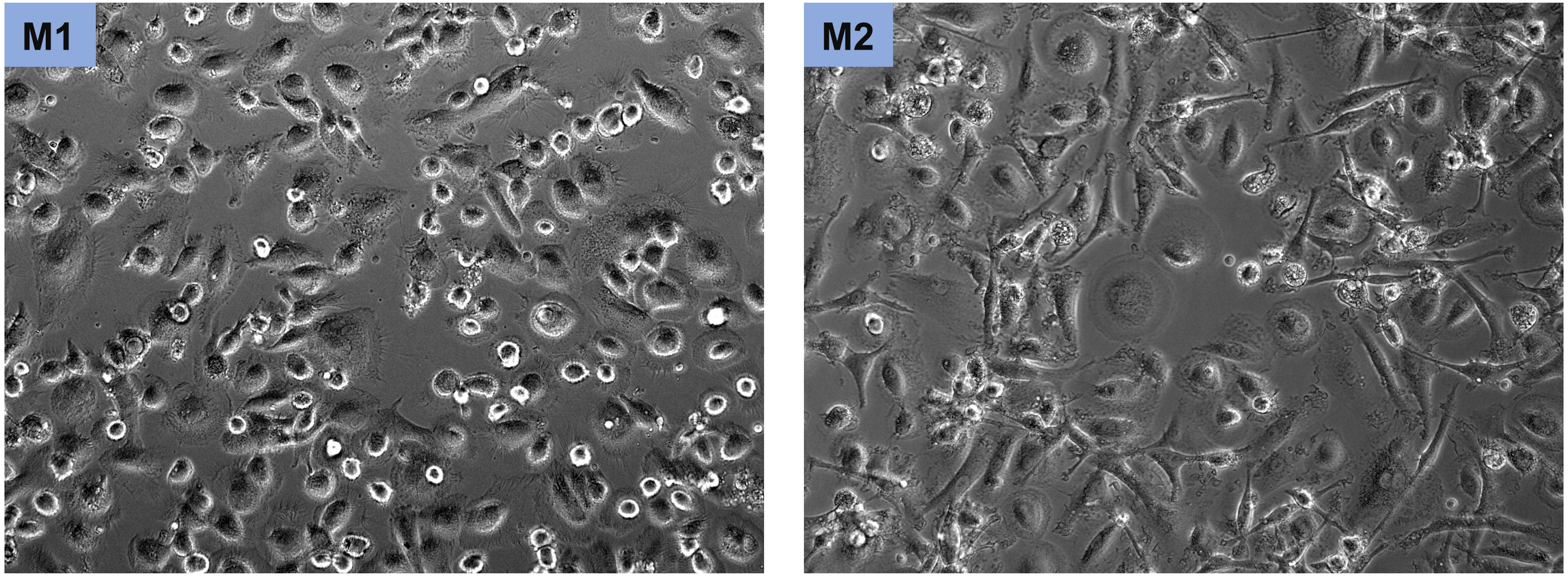


**Figure S2**. **Light microscopy of macrophages polarized into M1 and M2 phenotypes**.
M1 macrophages were obtained from PBMC by differentiation with GM-CSF (50 ng/ml) and polarization with LPS (10 ng/ml) and IFN-y (50 ng/ml). M2 macrophages were obtained from PBMC by differentiation with M-CSF (50 ng/ml) and polarization with IL-4 (20 ng/ml). Brightness/Contrast parameters were adjusted using ImageJ software to better demonstrate morphological characteristics of M1 and M2 macrophages.

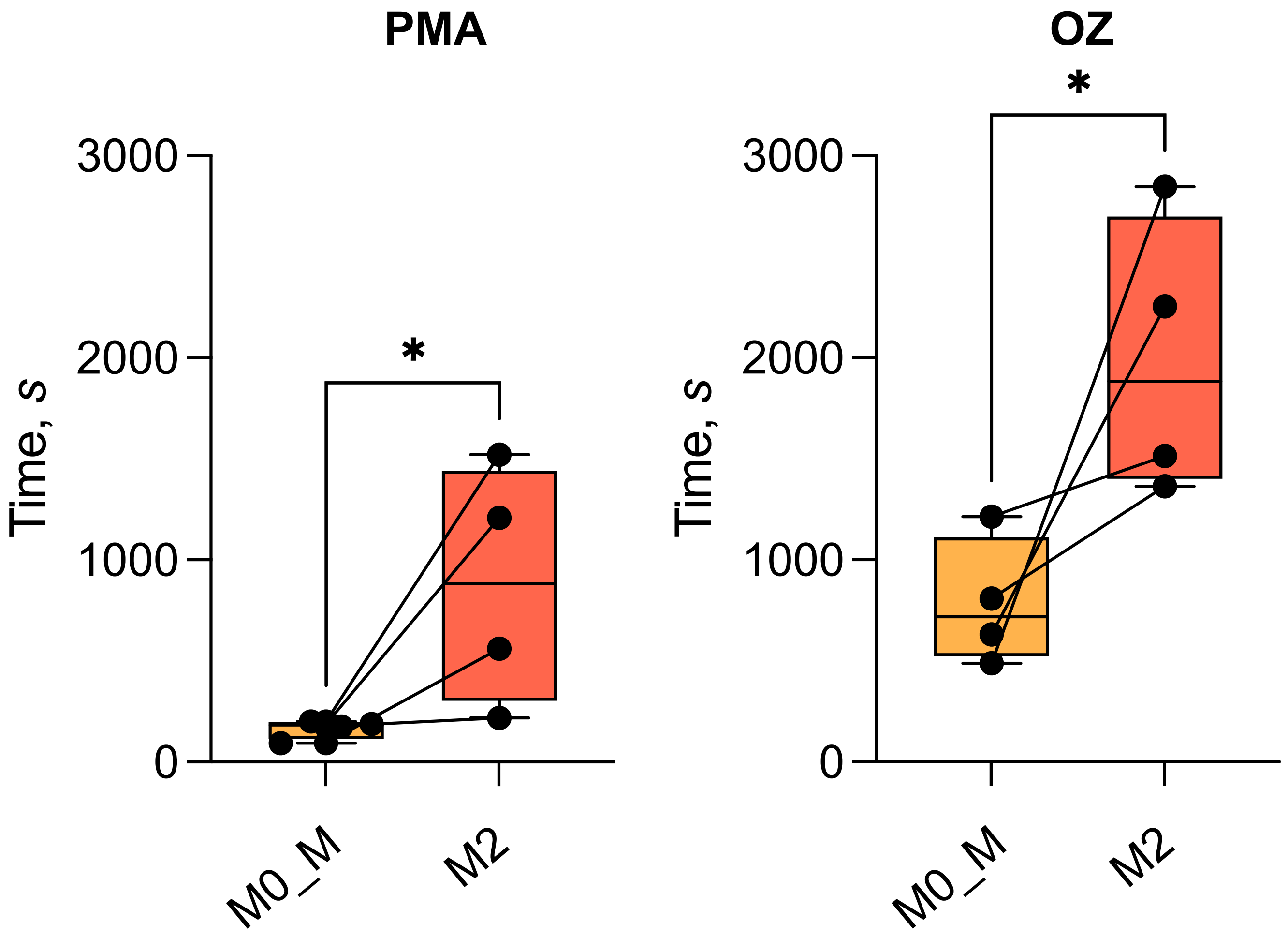


**Figure S3. CL peak time of activated M-CSF treated macrophages.**
CL of activated M2 macrophages reach maximal amplitude significantly later than CL of their precursors M0_M macrophages. Moreover, both M0_M and M2 macrophages reach CL maximum faster after activation with PMA than after activation with OZ. M0_M versus M2: *$P < 0.05$, Mann-Whitney U test, n = 4.

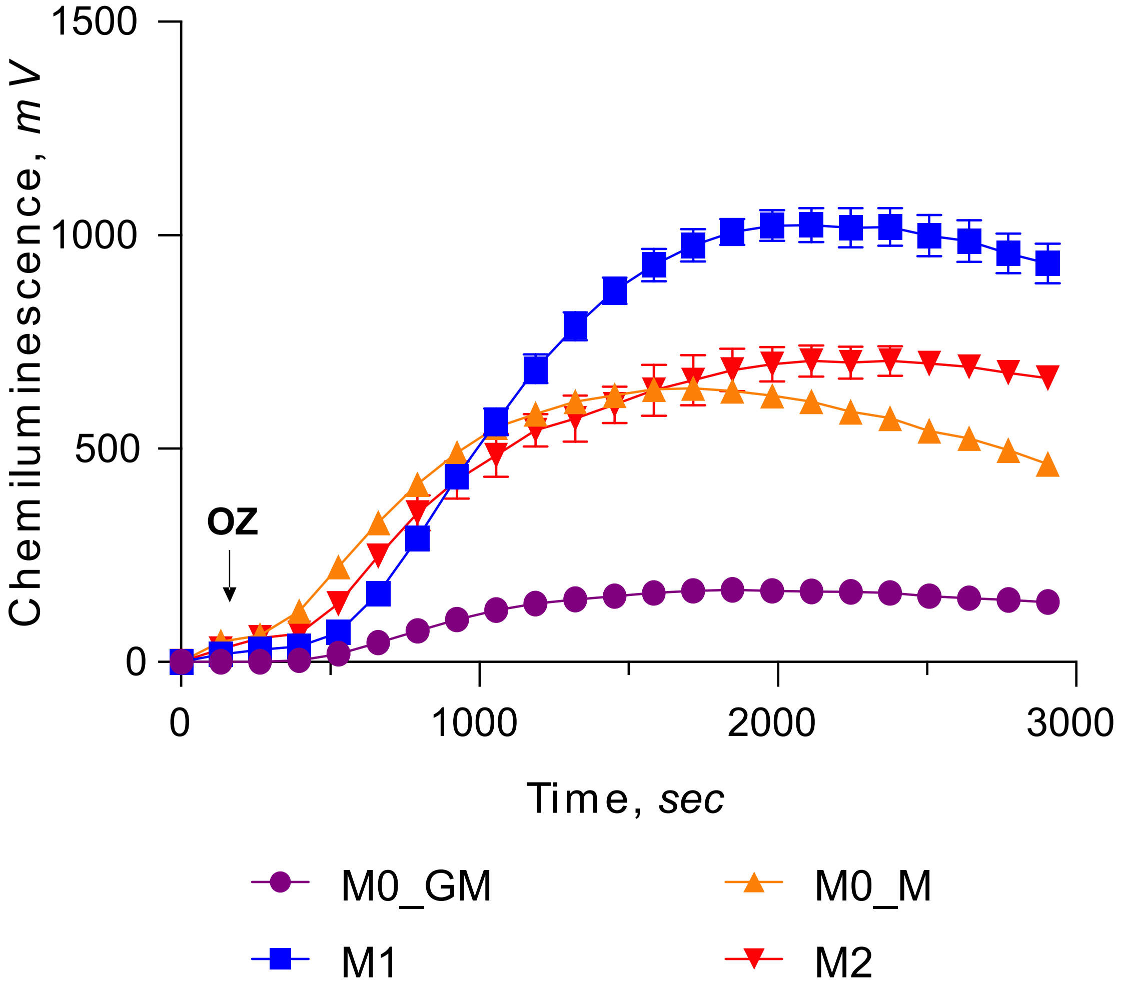


**Figure S4. ROS-generating activity of MDM in suspensions.**
Cells were detached using accutase solution and washed twice with PBS. Suspension of MDM in Krebs-Ringer buffer (with $NaHCO_3$ and $CaCl_2$) was placed to the cuvette of chemiluminometer. 430 μM luminol and 140 μg/ml HRP were added. The arrows indicate the time of addition of 300 μg/ml OZ. Measurements were performed at 37°C until the maximum was passed by all CL curves.

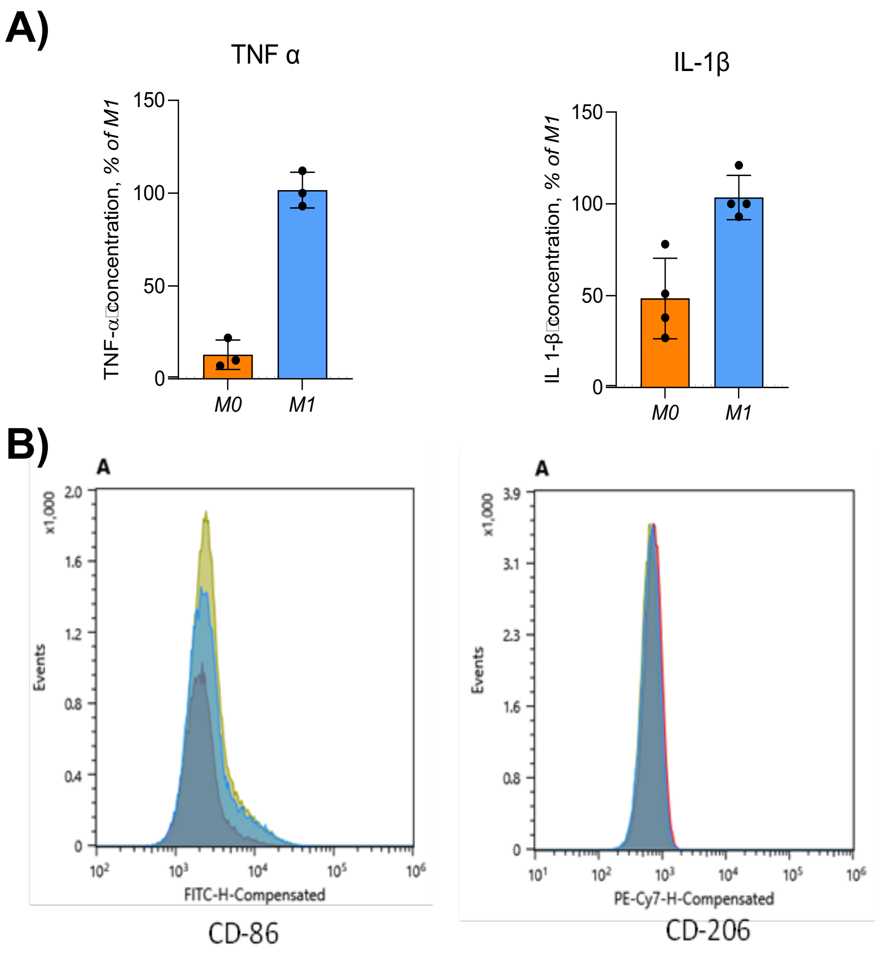


**THP-1 cells** (Merck, SCC223) were maintained in a glutamine-free RPMI-1640 medium (Gibco, Grand Island, New York, USA) supplemented with glutamax (2 mM), 10% fetal bovine serum and antibiotics (penicillin 100U/ml + streptomycin 100 mkg/ml) in a humidified atmosphere of 5% $CO_2$ and 95% air at 37°C. As the number of cells increased, they were distributed among the flasks so that the number of cells in the medium did not exceed 1 million/ml. To conduct experiments with THP-1 macrophages, cells were detached, resuspended in a medium and seeded into a 12-well plate with 0.45 million cells in 1.5 ml per well. Differentiation of cells into macrophages was achieved by incubation with phagocyte activator Phorbol 12-Myristate 13-Acetate (PMA, 50 ng/ml) within 48 hours. After 2 days without PMA, the polarization of macrophages in the M1 state was achieved by treating cells with IFN-gamma (20 ng/ml) and LPS (10 ng/ml), and in the M2 state with cytokine IL-4 and IL-13 (20 ng/ml each).

**Figure S5. THP-1 polarization markers.** (A) Amounts of cytokines TNF-α and IL-1b in conditioned media of macrophages measured by ELISA; (B) Flow cytometry histograms present fluorescence intensity of M0, M1, and M2 macrophages incubated with anti-CD86-FITC or anti-CD206-PE-Cy7 antibodies.
It is highly challenging to properly evaluate polarization of THP-1 state in M2 phenotype, as they don't express typical markers of M2 macrophages (CD206 and CD163) [43].

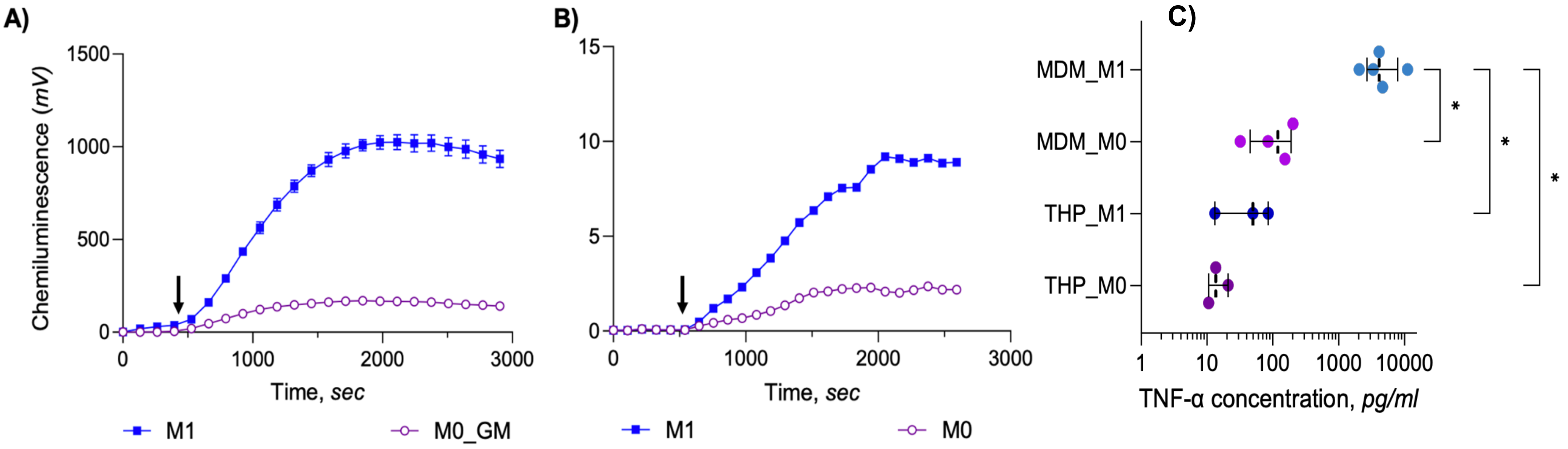


**Figure S6. Comparison of pro-inflammatory properties of MDM and THP1-macrophages.**
**A-B) ROS-generating activity measured with luminol-dependent chemiluminescence** (CL). Cells were deattached using accutase solution and washed twice with PBS. Suspension of MDM (A) or THP1-macrophages (B) in Krebs-Ringer buffer (with $NaHCO_3$ and $CaCl_2$) was added to the cuvette of chemiluminometer and 200 μM luminol. The arrows indicate the time of addition of 300 ug/ml OZ. Measurements for a given incubation time were carried out at 37$^{o}$C until the maximum was passed by all CL curves. The results are typical of two independent experiments.
**C) Secretion of TNF-α by THP1-macrophages and MDM.** Cytokine concentration was measured in supernatants collected after cultivation of THP-1 or MDM using ELISA kit to TNF-α. *$P < 0.05$, Analysis of variance (ANOVA) with Tukey's post hoc test.

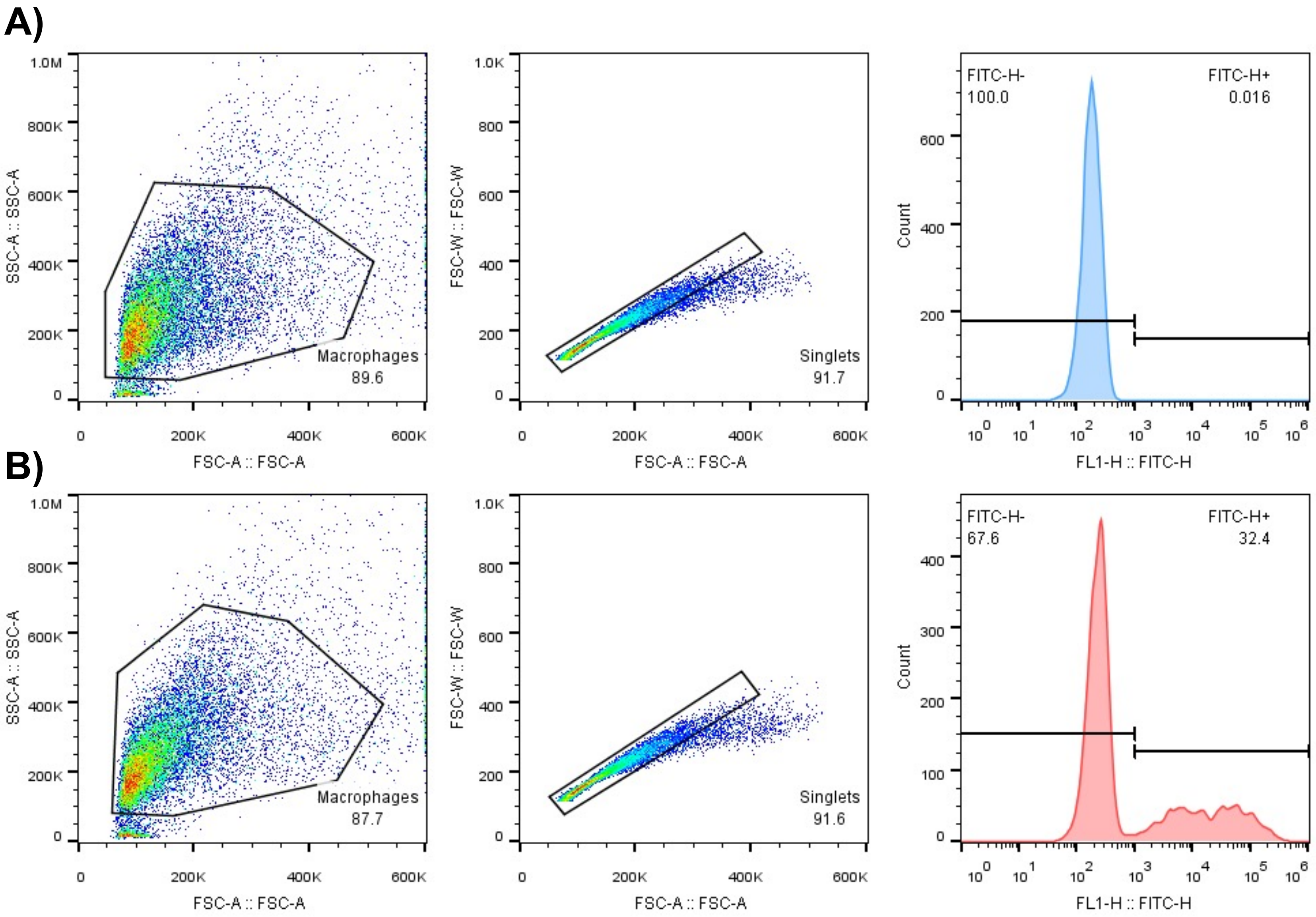


**Figure S7. Gating strategy used to identify phagocyting (FITC-positive) macrophages.** Unstained macrophages **(A)** and macrophages, incubated with FITC-conjugated BioParticles **(B)**. FSC-A versus SSC-A density plot was used to identify cells and exclude debris. FSC-W versus FSC-A density plot was performed to exclude doublets. Histograms represent FITC fluorescence intensity. Unstained control was used to separate phagocytic cells from non-phagocytic cells.

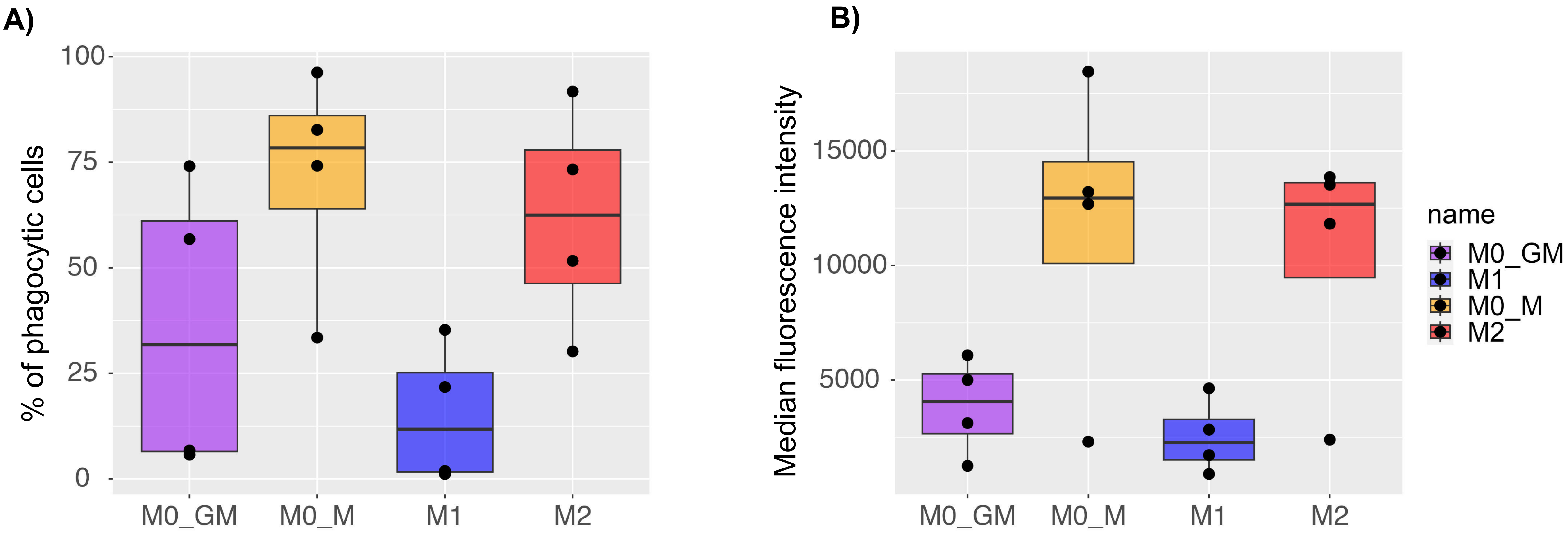


**Figure S8. Absolute values of phagocytic affinity and capacity**
Phagocytic affinity **(A)** and phagocytic capacity **(B)** of human macrophages, n = 4. Absolute values represent high variability.

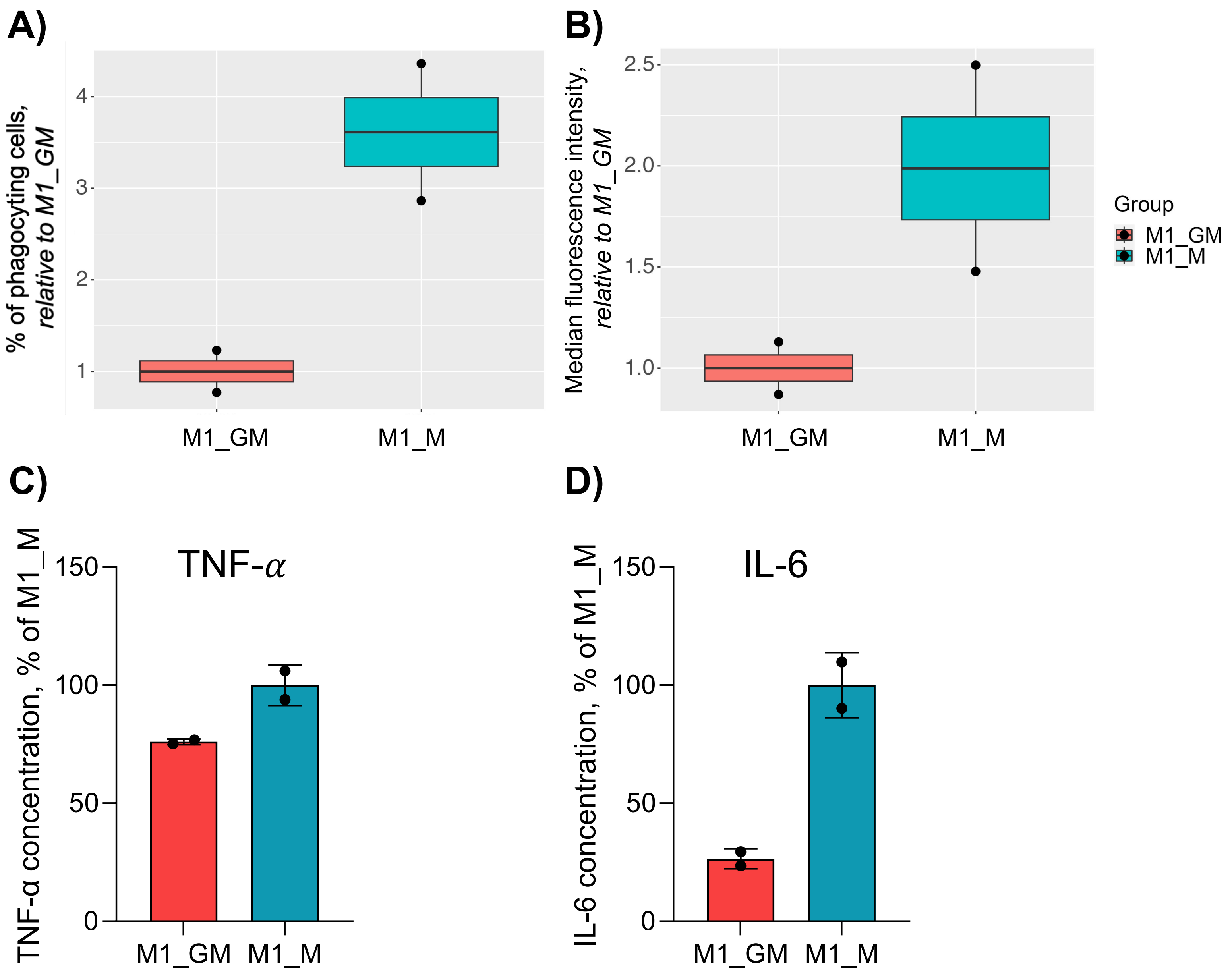


**Figure S9.**
Comparison of phagocytic affinity **(A),** phagocytic capacity **(B)** and pro-inflammatory TNF-a **(C)** and IL-6 **(D)** cytokines secretion by M1_M and M1_GM macrophages, n = 2.

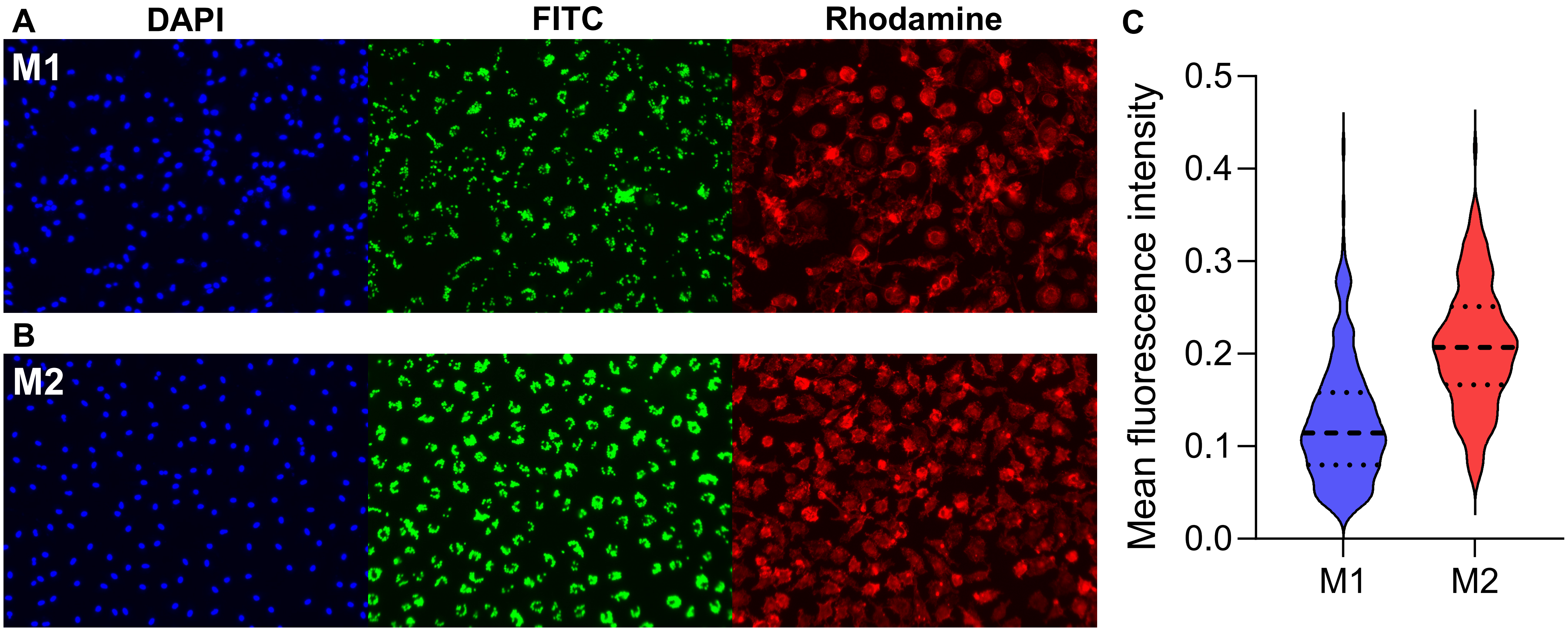


**Figure S10. Fluorescence microscopy of phagocyting M1 (A) and M2 (B) macrophages.** DAPI — nuclear staining; FITC — FITC-conjugate opsonized zymosan particles; Rhodamine — Rhodamine phalloidin for F-actin. **(C)** Mean fluorescence intensity of FITC per cell, representing phagocytic capacity of macrophages. The results are typical of two independent experiments.

OZ was suspended in 0.1 M sodium bicarbonate buffer pH 9.2 at concentration of 3 mg/ml. FITC diluted in ethanol was added to the protein solution to final concentration ~0.4 mg/ml and the mixture was incubated at room temperature for 4 h. OZ was spin down (3000rpm 15 min), washed with Henk's buffer, and finally diluted in Henk's at concentration of 6 mg/ml.

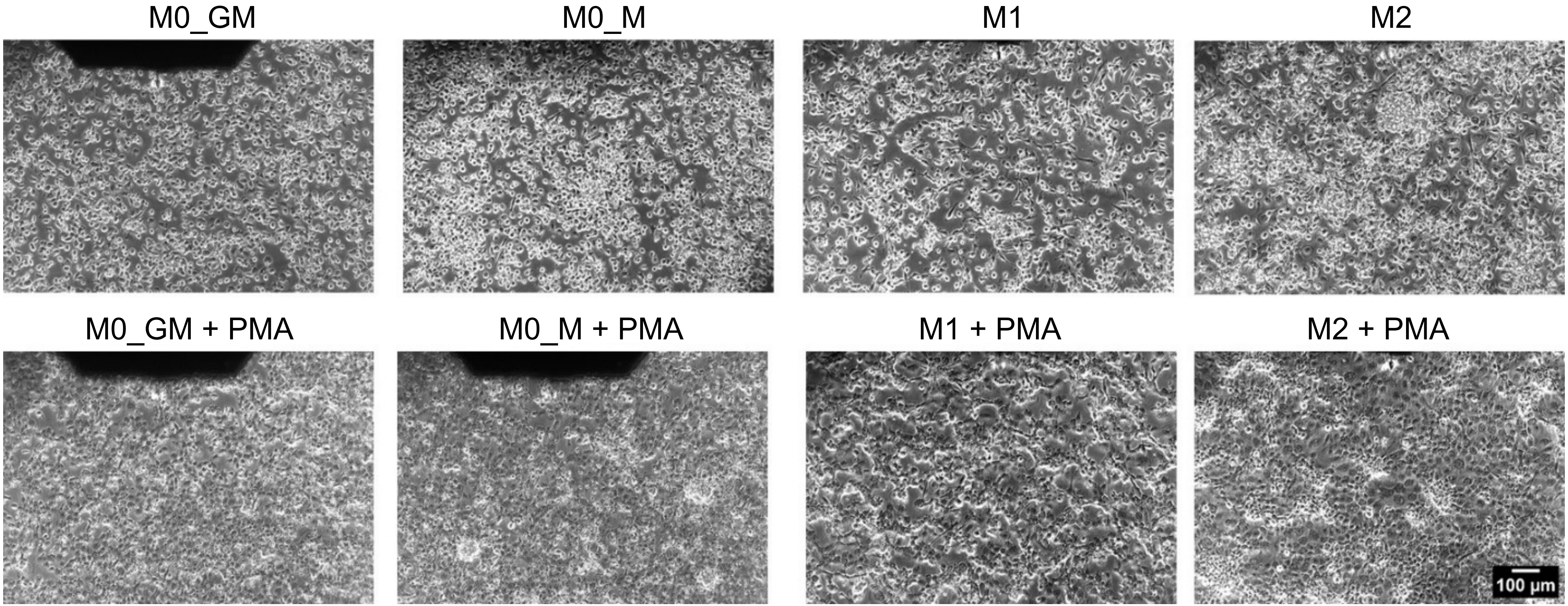


**Figure S11**. **Light microscopy of different groups of macrophages before and after activation with PMA.** Monocyte differentiation into macrophages and M0-macrophage polarization into M1 and M2 macrophages was performed as described in Material and Method section. Cells were incubated with PMA for 40 min, pictures were made prior to start AFM measurements. Concentration of PMA – 100 ng/ml.

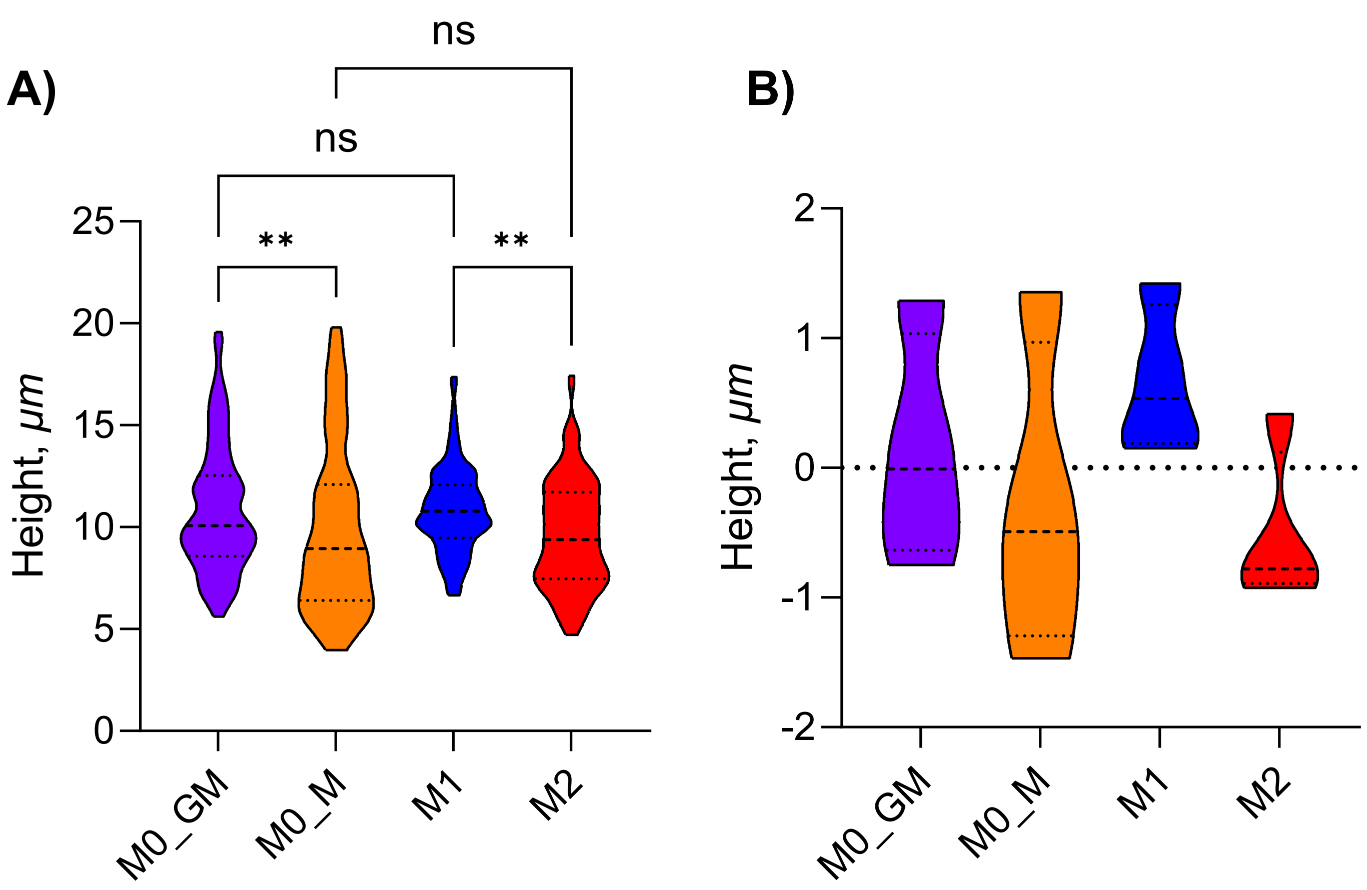


**Figure S12. Height of the untreated human macrophages in absolute values (A) and z-scaled (B).** For (A) Kruskal-Wallis one-way analysis of variance with Dunn's post-hoc test was performed to compare differences between groups. The analysis includes the results of 4 independent experiments, in each of which the studied parameters were measured for a minimum of 20 individual cells. $^{ns}P > 0.05$, $^{**}P < 0.01$.

A)

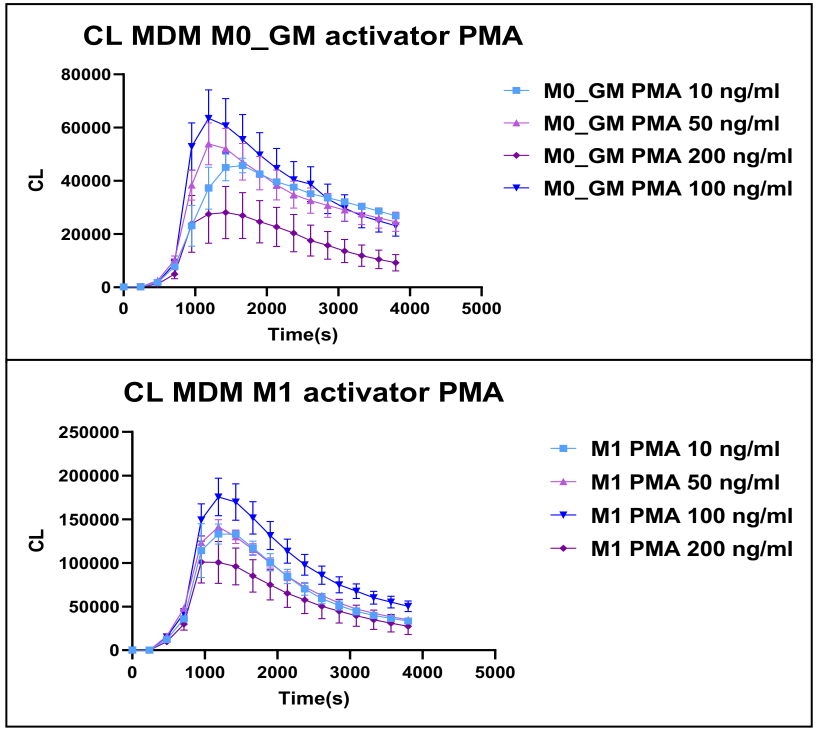


B)

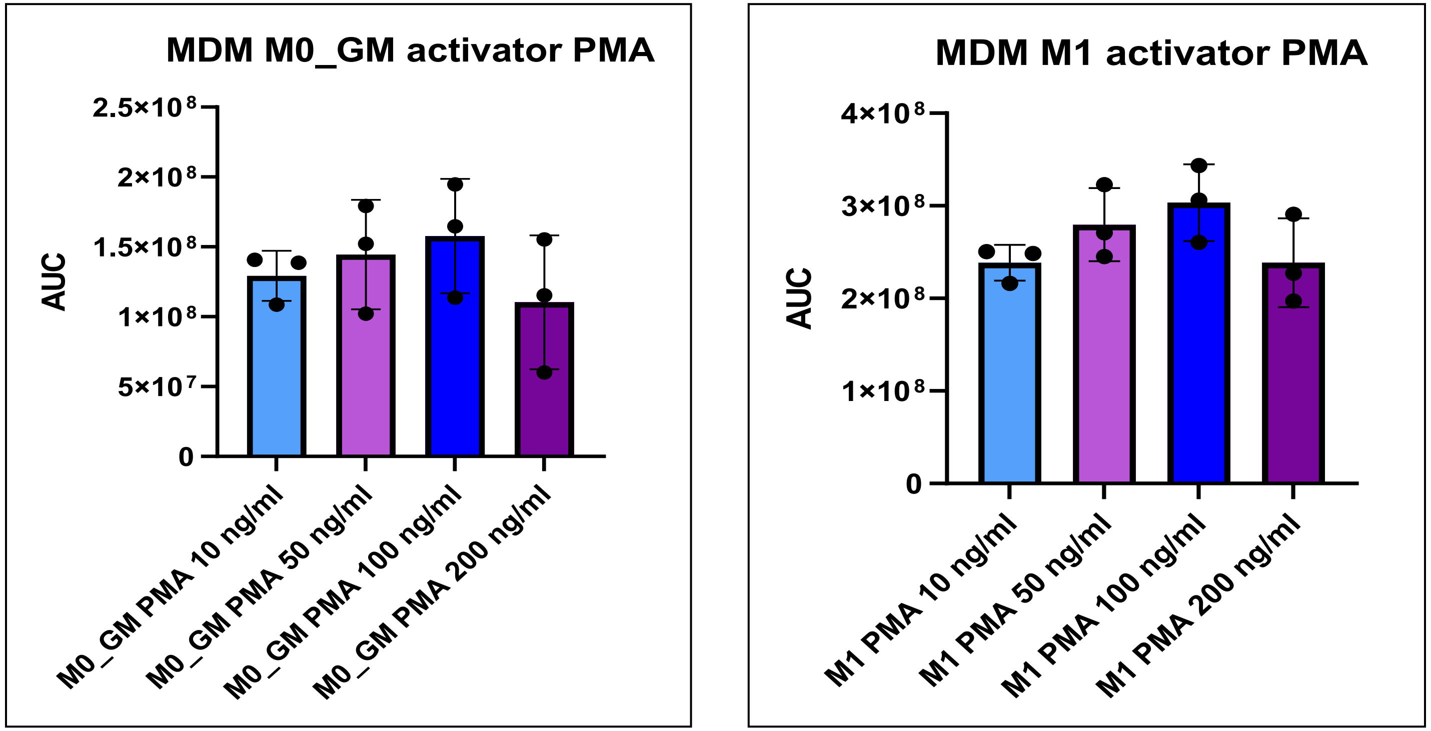


C)

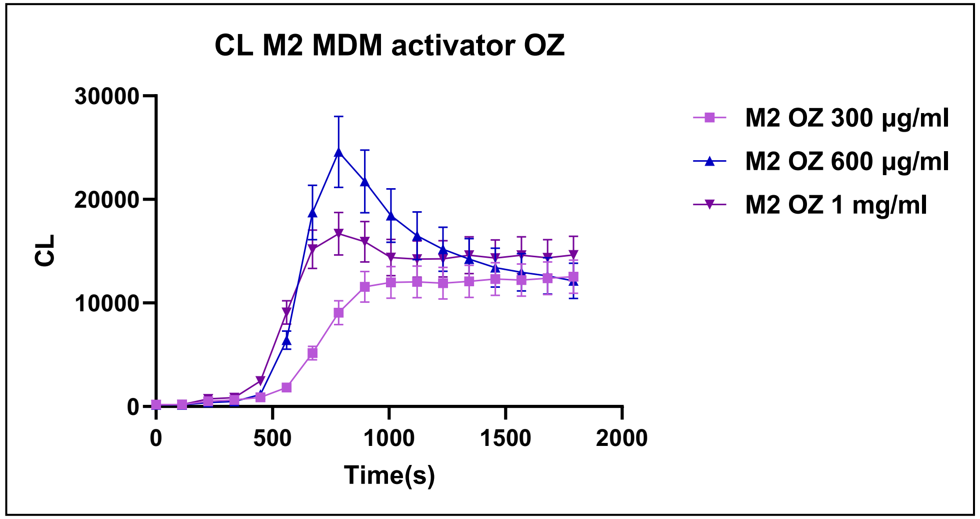


**Figure S13. ROS-generating activity of MDM treated with different concentrations of activator as assayed with luminol-dependent chemiluminescence (CL).**
**A, B)** Kinetics and comparison of CL of M0_GM and M1 macrophages treated with 10, 50, 100 and 200 ng/ml of PMA (AUC – area under curve). **C)** Radical-generated activity of M2 macrophages activated with 0.3, 0.6 and 1 mg/ml. Kinetics are typical of at least two independent experiments.

**Table S1.** Growth factor, cytokine concentrations and incubation times used for monocytes differentiation into macrophages and polarization into M1 and M2 states.

| Differentiation | CSF M1 | CSF M2 | LPS | IFN-y | IL-4 | IL-13 | Polarization | Reference | DOI |
|---|---|---|---|---|---|---|---|---|---|
| 6d | M-CSF 25 ng/mL | M-CSF 25 ng/mL | 100 ng/mL | 10 ng/mL | 20 ng/mL | - | 48h | Bertani et. al. 2017 | 10.1038/s41598-017-08121-8 |
| 6d | GM-CSF 50 ng/mL | M-CSF 50 ng/mL | 10 ng/mL | 50 ng/mL | 20 ng/mL | - | 18-20h | Mily et. al. 2020 | 10.3791/61807 |
| 6d | GM-CSF 50 ng/mL | M-CSF 50 ng/mL | 50 ng/mL | 20 ng/mL | 20 ng/mL | | 24h | Mia et. al. 2014 | 10.1111/sji.12162 |
| 6d | GM-CSF or M-CSF 25 ng/mL | | 10 ng/mL | 10 ng/mL | 10 ng/mL | - | - | Pilling et. al. 2017 | 10.1186/s12865-017-0214-z |
| 6d | GM-CSF 20 ng/mL | M-CSF 20 ng/mL | 50 ng/mL | 100 ng/mL | 20 ng/mL | 20 ng/mL | 24h | Lara-Reyna et. al. 2019 | 10.3389/fimmu.2019.01789 |
| 7d | GM-CSF 50 ng/mL | M-CSF 50 ng/mL | 100 ng/mL | 200 ng/mL | 40 ng/mL | - | 48h | Nzeteu et. al. 2022 | 10.3389/fimmu.2022.837097 |
| 7d | M-CSF 50 ng/mL | M-CSF 50 ng/mL | 100 ng/mL | 20 ng/mL | 25 ng/mL | - | 24h | Lewis et. al. 2019 | 10.1155/2019/1278301 |
| 7d | M-CSF 50 ng/mL | M-CSF 50 ng/mL | 100 ng/mL | 20 ng/mL | - | - | 24h | Scott et. al. 2023 | 10.1038/s41598-023-46237-2 |
